# Formulated Chinese medicine Shaoyao Gancao Tang reduces NLRP1 and NLRP3 in Alzheimer’s disease cell and mouse models for neuroprotection and cognitive improvement

**DOI:** 10.18632/aging.203125

**Published:** 2021-06-09

**Authors:** Ya-Jen Chiu, Chih-Hsin Lin, Ming-Chung Lee, Hsiu Mei Hsieh-Li, Chiung-Mei Chen, Yih-Ru Wu, Kuo-Hsuan Chang, Guey-Jen Lee-Chen

**Affiliations:** 1 Department of Life Science, National Taiwan Normal University, Taipei 11677, Taiwan; 2 Department of Neurology, Chang Gung Memorial Hospital, Chang Gung University College of Medicine, Taoyuan 33302, Taiwan; 3 Sun Ten Pharmaceutical Co. Ltd., New Taipei City 23143, Taiwan

**Keywords:** Alzheimer's disease, Aβ, anti-inflammation, neuroprotection, therapeutics

## Abstract

Amyloid β (Aβ) plays a major role in the neurodegeneration of Alzheimer’s disease (AD). The accumulation of misfolded Aβ causes oxidative stress and inflammatory damage leading to apoptotic cell death. Traditional Chinese herbal medicine (CHM) has been widely used in treating neurodegenerative diseases by reducing oxidative stress and neuroinflammation. We examined the neuroprotective effect of formulated CHM Shaoyao Gancao Tang (SG-Tang, made of *Paeonia lactiflora* and *Glycyrrhiza uralensis* at 1:1 ratio) in AD cell and mouse models. In Aβ-GFP SH-SY5Y cells, SG-Tang reduced Aβ aggregation and reactive oxygen species (ROS) production, as well as improved neurite outgrowth. When the Aβ-GFP-expressing cells were stimulated with conditioned medium from interferon (IFN)-γ-activated HMC3 microglia, SG-Tang suppressed expressions of inducible nitric oxide synthase (iNOS), NLR family pyrin domain containing 1 (NLRP1) and 3 (NLRP3), tumor necrosis factor (TNF)-α, interleukin (IL)-1β and IL-6, attenuated caspase-1 activity and ROS production, and promoted neurite outgrowth. In streptozocin-induced hyperglycemic APP/PS1/Tau triple transgenic (3×Tg-AD) mice, SG-Tang also reduced expressions of NLRP1, NLRP3, Aβ and Tau in hippocampus and cortex, as well as improved working and spatial memories in Y maze and Morris water maze. Collectively, our results demonstrate the potential of SG-Tang in treating AD by moderating neuroinflammation.

## INTRODUCTION

Alzheimer disease (AD) is the most common cause of dementia characterized by the presence of aberrant senile plaques in patients’ brain [1]. Senile plaques are composed of β amyloid peptide (Aβ), a proteolytic fragment of the amyloid beta precursor protein (APP) [2–4]. Aβ displays a neurotrophic support on differentiating neurons, but at the high concentration in mature neurons, as in AD, is neurotoxic [5]. Aβ oligomers or other high-order structures cause rapid influx of external calcium, oxidative stress and neuroinflammatory response, leading to apoptotic cell death [6, 7]. Treatment of AD is currently symptomatic, although trials aiming to reduce the production and burden of Aβ aggregation within the brain are underway [8, 9].

Inflammation has emerged as a central mechanism in AD and a potential therapeutic target for treatment [10]. Studies have demonstrated that Aβ aggregation-linked neuroinflammation causes neuronal damage and clinical deterioration. Microglia, a group of highly motile phagocytes in central nervous system and frequently found in close proximity to Aβ aggregates in AD patients [11, 12], could be activated by Aβ [13]. Aβ binds to several innate immune receptors present on microglia, such as Toll-like receptor 2 (TLR2), TLR4 and TLR6 [14, 15], all of which can activate microglia. Microglial activation increases the production of pro-inflammatory factors, such as tumor necrosis factor (TNF)-α, interleukin (IL)-1β, IL-6, nitric oxide (NO) produced by inducible nitric oxide synthase (iNOS), and reactive oxygen species (ROS) [16, 17]. Furthermore, inflammasomes, such as NLR family pyrin domain containing 1 (NLRP1) and 3 (NLRP3), are also activated in brains of patients with AD [18]. These observations strongly suggest that neuroinflammation plays a crucial role in the pathogenesis of AD.

Lines of evidence suggest that herb medicine can reduce neuroinflammation, and thus could be a treatment for AD. For example, *Oenanthe javanica* has various pharmacological and biological activities such as anti-inflammatory [19] and anti-oxidative [20] activities. Extract of *Flemingia philippinensis* contains various isoflavones, which exhibit anti-oxidative and anti-inflammatory activities [21, 22]. Shaoyao Gancao Tang (SG-Tang), a formulated Chinese herbal medicine (CHM) made of *Paeonia lactiflora* (*P. lactiflora*) and *Glycyrrhiza uralensis* (*G. uralensis*), displays anti-oxidative and anti-inflammatory activities for neuroprotection in neurodegenerative cell models [23]. The integrative pharmacology approach also discloses the therapeutic mechanisms of Danggui-Shaoyao-san decoction, which is a formulation of BaiShao, DangGui, BaiZhu, ChuanXiong, ZeXie and FuLing, against AD [24]. In addition, SG-Tang can reduce neuronal TBP aggregation and exert neuronal protection in spinocerebellar ataxia cell and mouse models [25]. A network pharmacology-based study further discloses the active compounds and therapeutic targets of SG-Tang in Parkinson’s disease (PD) [26]. As Aβ is a validated target for developing therapeutic agents, we evaluated the potential of SG-Tang against Aβ-aggregation and neuroinflammation by our established Aβ-GFP-expressing SH-SY5Y cell model [27], and triple-transgenic AD mouse model harboring APP_Swe_, PS1_M146V_, and Tau_P301L_ [28]. The results showed the potential of SG-Tang to mitigate Aβ-mediated neurotoxicity and neuroinflammation, providing a new drug candidate in treating AD.

## RESULTS

### Aβ aggregation inhibition and neurite outgrowth promotion of SG-Tang

**Figure 1:**
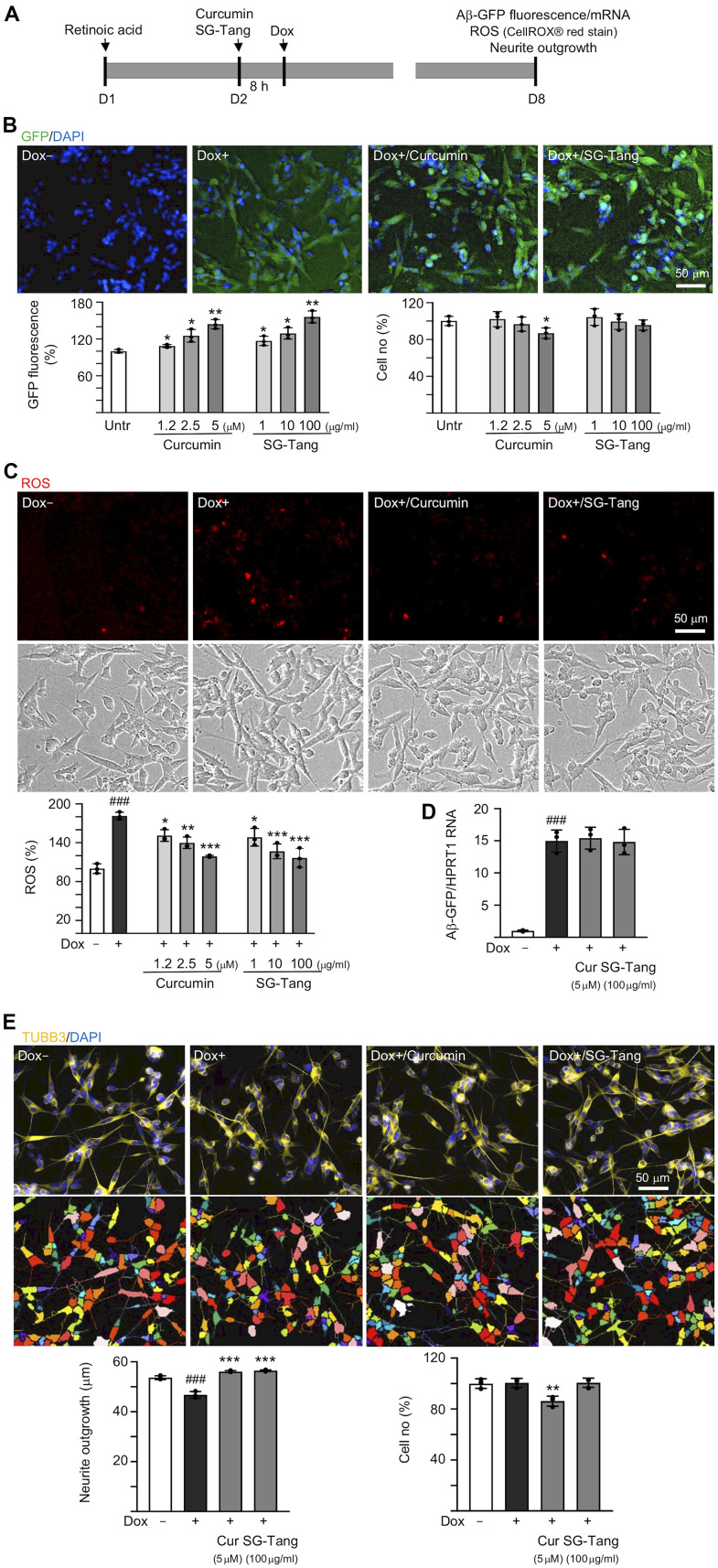
**Effects of SG-Tang on Aβ aggregation, ROS, and neurite outgrowth in Aβ-GFP-expressing cells.** (**A**) Experimental flow chart of Aβ-GFP SH-SY5Y cells. On day 1, cells were plated with retinoic acid (RA, 10 μM) added to the culture medium. On day 2, curcumin or SG-Tang was added to the cells for 8 h, followed by inducing Aβ-GFP expression with doxycycline (Dox, 5 μg/ml) for 6 days. On day 8, GFP fluorescence, cell number, ROS, Aβ-GFP RNA and neurite outgrowth were measured. (**B**) Assessment of GFP fluorescence and cell number with curcumin (1.2–5 μM) or SG-Tang (1–100 μg/ml) treatment (*n* = 3). The relative GFP fluorescence/cell number of untreated cells (Untr.) was normalized as 100%. (**C**) ROS assay with curcumin (1.2–5 μM) or SG-Tang (1–100 μg/ml) treatment (*n* = 3). The relative ROS of uninduced cells (Dox-) was normalized (100%). (**D**) Measurement of Aβ-GFP RNA levels in cells treated with 5 μM curcumin and 100 μg/ml SG-Tang by real-time PCR (n = 3). (**E**) Fluorescence microscopy images of differentiated Aβ-GFP SH-SY5Y cells uninduced (Dox-), untreated (Dox+) or treated with curcumin (5 μM) or SG-Tang (100 μg/ml). Neurite outgrowth and cell number were measured after TUBB3 (yellow) staining (n = 3). Nuclei were counterstained with DAPI (blue). The relative cell number of uninduced cells was normalized as 100%. *P* values: comparisons between induced (Dox+) vs. uninduced (Dox-) cells (^###^: *P* < 0.001), or treated (Dox+/curcumin or SG-Tang) vs. untreated (Dox+) cells (*: *P* < 0.05, **: *P* < 0.01, ***: *P* < 0.001). (B: GFP fluorescence and cell number: two-tailed Student’s *t* test; C–E: ROS, Aβ-GFP RNA and neurite outgrowth: one-way ANOVA with a *post hoc* Tukey test).

In order to evaluate how effective SG-Tang can be in its use against Aβ aggregation, we treated retinoic acid-differentiated Aβ-GFP-expressing SH-SY5Y cells with different concentrations of SG-Tang (Figure 1A). In this cell model, the level of Aβ misfolding was negatively correlated with GFP fluorescence intensity [29]. The 1.2–5 μM curcumin treatment increased the green fluorescence intensity (109–144%, *P* = 0.043–0.005; cell viability: 102−87%). Treatments with SG-Tang at 1–100 μg/ml also increased the green fluorescence intensity (117–156%, *P* = 0.048–0.006; cell viability: 104−96%) (Figure 1B). In the analysis of oxidative stress, overexpression of Aβ also elevated the ROS level (181%, *P* < 0.001), while treatment with curcumin (1.2–5 μM) or SG-Tang (1–100 μg/ml) effectively mitigated the increased ROS (from 181% to 151–117%, *P* = 0.012–<0.001) (Figure 1C). Meanwhile, treatment of curcumin at 5 μM or SG-Tang at 100 μg/ml did not affect Aβ-GFP RNA level (*P* > 0.05) (Figure 1D), suggesting that SG-Tang may improve Aβ-GFP protein misfolding without affecting gene expression.

The neuroprotective effect of SG-Tang was then evaluated by Tet-On Aβ-GFP SH-SY5Y cells. Overexpression of Aβ decreased neurite outgrowth (from 54 μm to 47 μm, *P* = 0.004). The pre-treatment with curcumin (5 μM) or SG-Tang (100 μg/ml) effectively rescued this impairment of neurite outgrowth (from 47 μm to 56 μm, *P* < 0.001) (Figure 1E). During the 7-day incubation of SG-Tang, there wasn’t any toxic effect on cell survival (101% for 100 μg/ml treatment). However, the cytotoxicity increased slightly for curcumin at 5 μM concentration (cell viability 86%, *P* = 0.007) in Aβ-GFP SH-SY5Y cells.

### IFN-γ-induced activation of human HMC3 microglia

**Figure 2:**
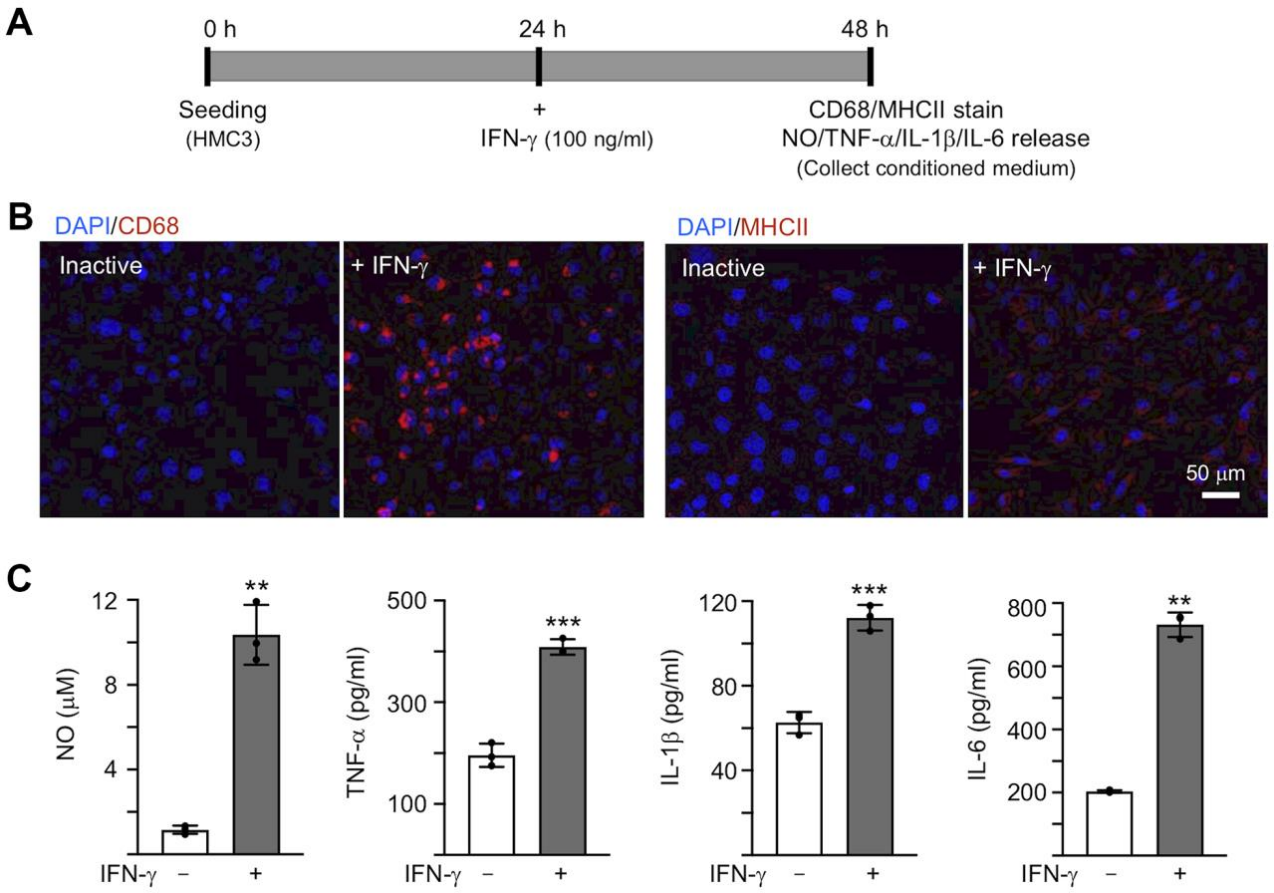
**IFN-γ-induced HMC3 activation.** (**A**) Experimental flow chart. After seeding HMC3 for 24 h, IFN-γ (100 ng/ml) was added to cells to induce inflammation. After 24 h, CD68 and HMCII expression in cells as well as NO, TNF-α, IL-1β and IL-6 release in culture medium were assessed. (**B**) Immunofluorescence examination of IFN-γ-induced HMC3 activation using antibodies against CD68 and HMCII (red). Cell nuclei were counterstained with DAPI (blue). (**C**) Levels of NO (measured by Griess reagent), TNF-α, IL-1β and IL-6 (assessed by ELISA) in culture medium (n = 3). *P* values: comparisons between IFN-γ-activated vs. inactive cells (**: *P* < 0.01, ***: *P* < 0.001). (two-tailed Student’s *t* test).

To activate microglia, 100 ng/ml IFN-γ was applied to human HMC3 microglial cells for 24 h [30] (Figure 2A). IFN-γ treatment increased expression of markers for microglial activation, such as CD68 and MHCII (Figure 2B). The production of NO in the culture medium was significantly increased by IFN-γ treatment (from 1.2 μM to 10.4 μM, *P* = 0.007), accompanying with increased levels of TNF-α (from 196.1 pg/ml to 408.6 pg/ml, *P* < 0.001), IL-1β (from 62.7 pg/ml to 112.3 pg/ml, *P* < 0.001) and IL-6 (from 203.9 pg/ml to 732.1 pg/ml, *P* = 0.002) (Figure 2C). In the following experiment, in order to trigger the neuroinflammation in Aβ-GFP SH-SY5Y cells, we used the HMC3 conditioned medium activated by IFN-γ (CM/+IFN-γ).

### Effects of SG-Tang on conditioned medium-stimulated Aβ-GFP SH-SY5Y cells

**Figure 3:**
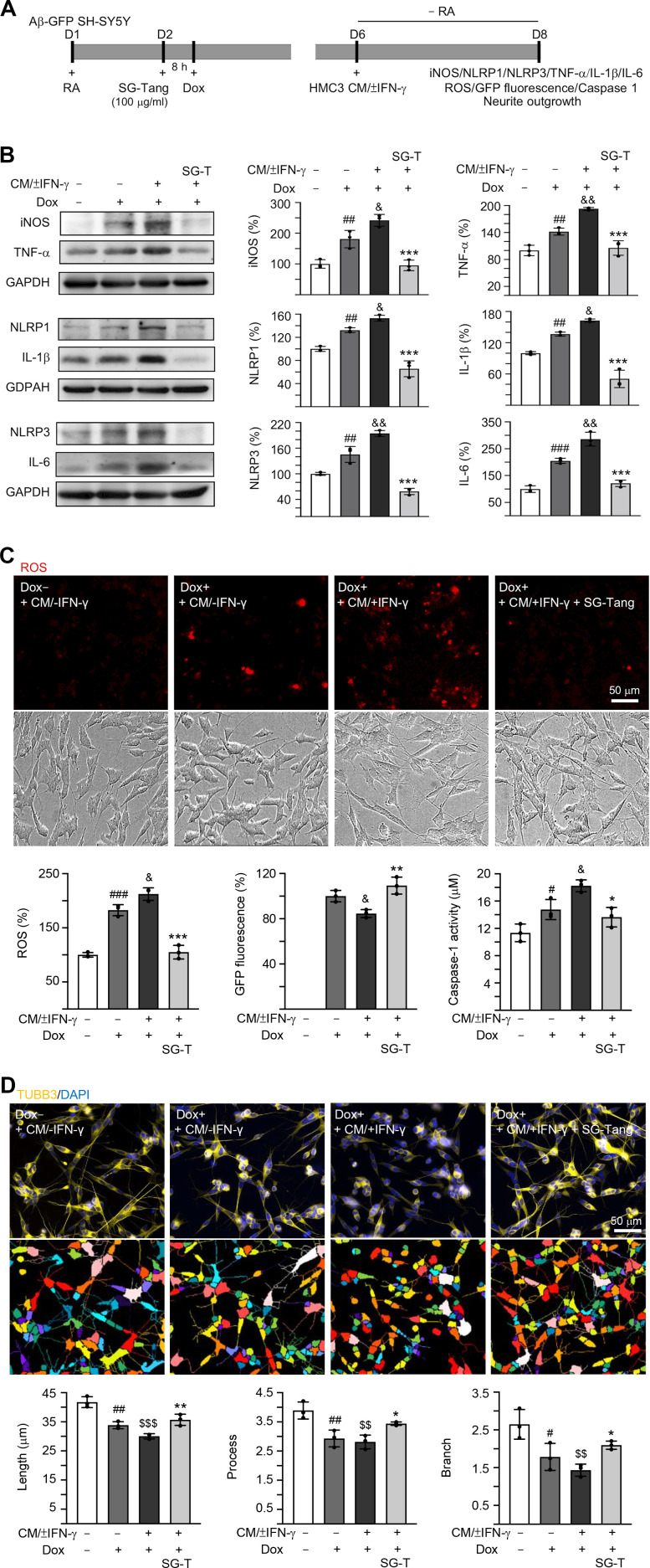
**Neuroprotective effect of SG-Tang in Aβ-GFP-expressing SH-SY5Y cells inflamed with HMC3 conditioned medium.** (**A**) Experimental flow chart. Aβ-GFP SH-SY5Y cells were plated in medium with retinoic acid (RA, 10 μM) on day 1, and treated with SG-Tang (100 μg/ml) next day for 8 h, followed by doxycycline (Dox, 5 μg/ml) addition to induce Aβ-GFP expression. On day 6, DMEM-F12 medium without retinoic acid addition (− RA) was mixed with HMC3 conditioned medium (CM/±IFN-γ, 1:1 ratio) and added to the cells for 2 days. (**B**) iNOS, NLRP1, NLRP3, TNF-α, IL-1β and IL-6 levels, (**C**) ROS production, GFP fluorescence and caspase-1 activity, (**D**) neurite length, process and branch were assessed on day 8 (n = 3). For normalization, the relative iNOS, NLRP1, NLRP3, TNF-α, IL-1β, IL-6 and ROS levels in uninduced and CM/-IFN-γ stimulated cells were set as 100%. *P* values: comparisons between induced (Dox+, + CM/-IFN-γ) vs. uninduced (Dox-,+ CM/-IFN-γ) cells (^#^: *P* < 0.05, ^##^: *P* < 0.01, ^###^: *P* < 0.001), induced and inflamed (Dox+, + CM/+IFN-γ) vs. induced (Dox+, + CM/-IFN-γ) cells (^&^: *P* < 0.05, ^&&^: *P* < 0.01), induced and inflamed (Dox+, + CM/+IFN-γ) vs. uninduced (Dox-, + CM/-IFN-γ) cells (^$$^: *P* < 0.01, ^$$$^: *P* < 0.001), or SG-Tang treated (+ CM/+IFN-γ, +SG-Tang) vs. untreated (+ CM/+IFN-γ) cells (*: *P* < 0.05, **: *P* < 0.01, ***: *P* < 0.001). (One-way ANOVA with a *post hoc* Tukey test).

In AD brains, neurons are exposed to high levels of pro-inflammatory cytokines, for example, TNF-α, IL-1β and IL-6 [31]. To model this neuroinflammatory niche, we applied CM/±IFN-γ to neuronal cells differentiated from Aβ-GFP SH-SY5Y cells for two days (Figure 3A). As shown in Figure 3B, the Aβ overexpression in SH-SY5Y cells increased the expressions of iNOS (181%, *P* = 0.006), NLRP1 (132%, *P* = 0.005), NLRP3 (145%, *P* = 0.004), TNF-α (142%, *P* = 0.007), IL-1β (137%, *P* = 0.004) and IL-6 (205%, *P* < 0.001). These up-regulations were further exaggerated after treating cells with CM/+IFN-γ (iNOS: 242%, NLRP1: 153%, NLRP3: 195%, TNF-α: 193%, IL-1β: 163%, IL-6: 286%; *P* = 0.049–0.001), whereas treatment with SG-Tang at 100 μg/ml normalized the levels of these markers for inflammasome and neuroinflammation pathways (iNOS: 95%, NLRP1: 66%, NLRP3: 59%, TNF-α: 106%, IL-1β: 51%, IL-6: 121%; *P* < 0.001) (Figure 3B). Furthermore, application of CM/+IFN-γ reduced GFP fluorescence (85%, *P* = 0.031), whereas SG-Tang treatment increased GFP fluorescence (109%, *P* = 0.004), reflecting the improvement of Aβ-GFP folding. Consistently, the increased ROS and caspase-1 activity in CM/+IFN-γ-treated Aβ-GFP SH-SY5Y cells were reduced by the treatment with SG-Tang (ROS level: from 212% to 105%, *P* < 0.001; caspase-1 activity: from 18 μM to 14 μM, *P* = 0.010) (Figure 3C). Moreover, the neurite total length (from 42 μm to 30 μm, *P* < 0.001), process (primary neurite, a projection from the cell body of a neuron; from 3.9 to 2.8, *P* = 0.003) and branch (an extension from primary neurite; from 2.6 to 1.4, *P* = 0.002) of the differentiated neuronal cells were also reduced by overexpression of Aβ and CM/+IFN-γ treatment, whereas SG-Tang rescued these impairments (neurite length: from 30 μm to 36 μm, *P* = 0.009; process: from 2.8 to 3.4, *P* = 0.049; branch: from 1.4 to 2.1, *P* = 0.047) (Figure 3D). These results show that SG-Tang could down-regulate inflammasome and neuroinflammation pathways. Moreover, it could reduce ROS production and caspase-1 activity, as well as improve neurite outgrowth in Aβ-GFP-expressing SH-SY5Y cells inflamed with CM/+IFN-γ.

### Effects of SG-Tang on spatial learning and memory impairments in 3×Tg-AD mice

**Figure 4:**
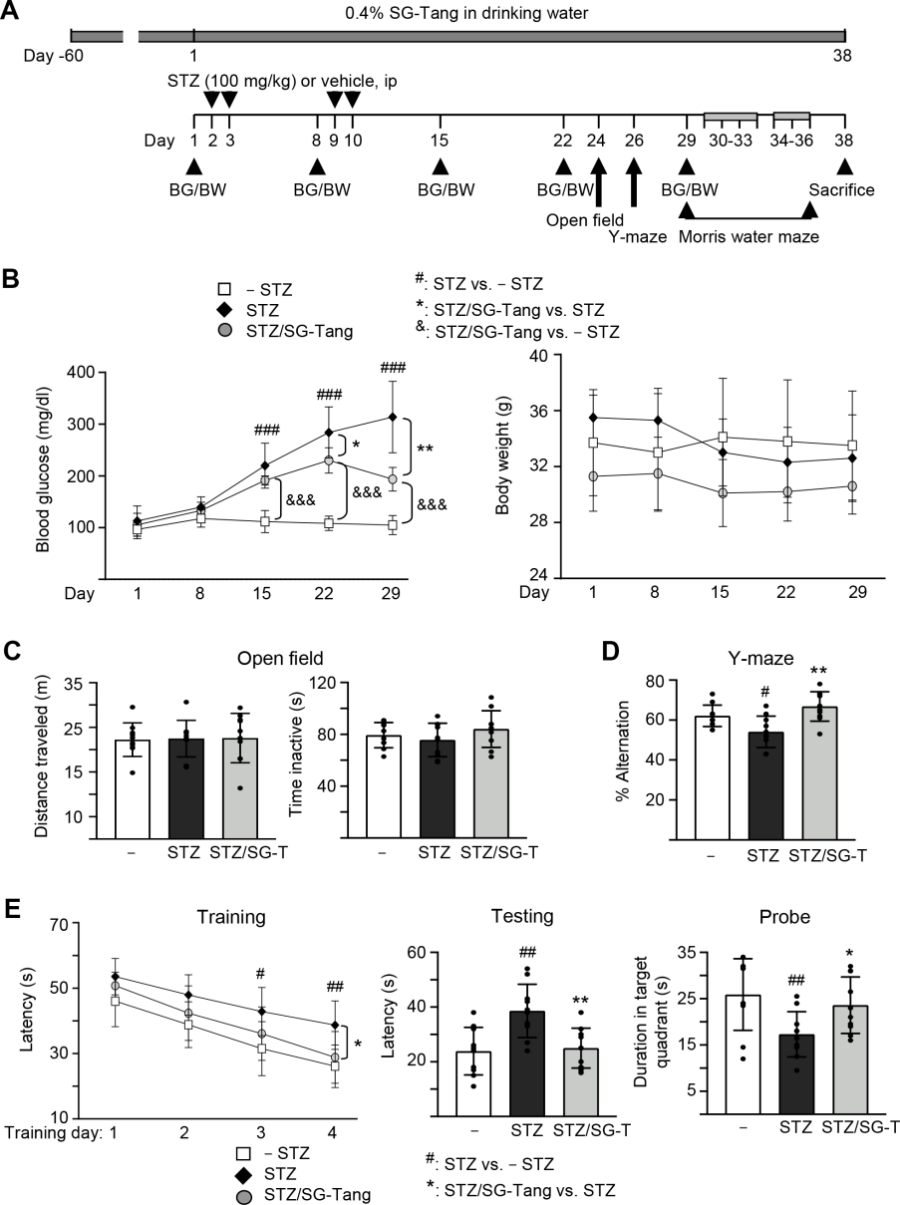
**Cognitive improvement of SG-Tang in STZ-treated 3×Tg-AD mice.** (**A**) Experimental flow chart. Mice received vehicle or SG-Tang (0.4% in drinking water) from day -60 to day 38. Blood glucose (BG) and body weight (BW) and were measured on days 1, 8, 15, 22 and 29. Mice were i.p. injected by streptozocin (STZ, 100 mg/kg) or vehicle (0.1 M sodium citrate pH4.5) at days 2, 3, 9 and 10. Open field, Y-maze and Morris water maze tasks were performed on days 24, 26 and 29–36, respectively. Mice in – STZ, STZ and STZ/SG-Tang groups (**B**–**E**) received vehicle, STZ and STZ+SG-Tang, respectively during the course of the experiment. (**B**) Body weight and blood glucose of the mice. (**C**) Open field measurement of motor activities (distance traveled) and anxious mood (time inactive) in 10 min of testing period. (**D**) Y-maze measurement of spontaneous alternation rate in 8 min of testing period. (E) Morris water maze testing of latency to find the hidden platform (latency) in training and testing and duration in target quadrant in probe trials. *P* values: comparisons between STZ vs. – STZ mice (^#^: *P* < 0.05, ^##^: *P* < 0.01, ^###^: *P* < 0.001), STZ/SG-Tang vs. STZ mice (*: *P* < 0.05, **: *P* < 0.01), or STZ/SG-Tang vs. – STZ mice (^&&&^: *P* < 0.001). (One-way or two-way ANOVA with a *post hoc* Tukey test).

We then used 3×Tg-AD mice to further explore the neuroprotective potential of SG-Tang *in vivo*. The homozygous 3×Tg-AD mice display diffuse amyloid plaques in the neocortex and Aβ aggregation in pyramidal neurons of the hippocampus, cortex and amygdale, and demonstrate trivial deficits in Morris water maze at 6 months of age [28, 32], while STZ-induced hyperglycemia greatly exacerbates the development of AD phenotypes [33]. Therefore, we injected STZ intraperitoneally into 6-month-old 3×Tg-AD mice (Figure 4A). As shown in Figure 4B, the injection of STZ increased blood glucose significantly, from 113 mg/dl (day 1) to 220–314 mg/dl (days 15–29, *P* < 0.001) in STZ group. Repeated measures of two-way ANOVA displayed a significant effect of day (*F* = 83.44, *P* < 0.001) and treatment (*F* = 212.4, *P* < 0.001) on blood glucose. A significant treatment × day interaction (*F* = 18.56, *P* < 0.001) was also found. Even though SG-Tang treatment reduced blood glucose on days 22–29 (from 284–314 mg/dl to 230–193 mg/dl, *P* = 0.040–0.002), the blood glucose levels in STZ/SG-Tang group remained significantly increased (191–230 mg/dl) in comparison to the normoglycemic group (– STZ, 105–112 mg/dl) (*P* < 0.001) on days 15–29. There wasn’t any significant change of body weight was observed among groups. Open field test performed on day 24 did not show any significant changes in travelled distance and inactive time of mice with STZ/SG-Tang treatment (Figure 4C). Y-maze alternation rate, which evaluated the working memory, was reduced in STZ group compared to control group (– STZ) (54% vs. 62%, *P* = 0.039), while SG-Tang treatment improved the alternation rates (from 54% to 67%, *P* = 0.001) (Figure 4D).

In order to evaluate the effect of SG-Tang on spatial learning and memory, we performed Morris water maze task in different phases: training (day 30–33), testing (day 34) and probe (day 36) trials. As shown in Figure 4E, the latency to locate the hidden platform was relatively longer in STZ group in comparison to the control group (– STZ) on training day 3 (43 s vs. 31 s, *P* = 0.014) and day 4 (39 s vs. 26 s, *P* = 0.007), whereas SG-Tang treatment reduced the latency on STZ-treated mice on training day 4 (from 39 s to 29 s, *P* = 0.011). Repeated measures of ANOVA disclosed a significant effect of day (*F* = 92.24, *P* < 0.001) and treatment (*F* = 7.098, *P* = 0.0096) on the latency without significant treatment × day interaction (F = 1.231, *P* = 0.3161). Testing trial also showed longer latency in STZ treated mice compared to normal control (– STZ) (39 s vs. 24 s, *P* = 0.002), whereas SG-Tang treatment consistently reduced the latency (from 39 s to 25 s, *P* = 0.004). In probe trial, the STZ-treated mice spent less time in the target quadrant than normal control (– STZ) (17 s vs. 26 s, *P* = 0.002). SG-Tang treatment increased the time spent in the target quadrant (from 17 s to 23 s, *P* = 0.022). These results show that SG-Tang has a positive impact on the working and spatial memories for the STZ-treated 3×Tg-AD mice.

### SG-Tang reduced Aβ and Tau levels in STZ-treated 3×Tg-AD mice

**Figure 5:**
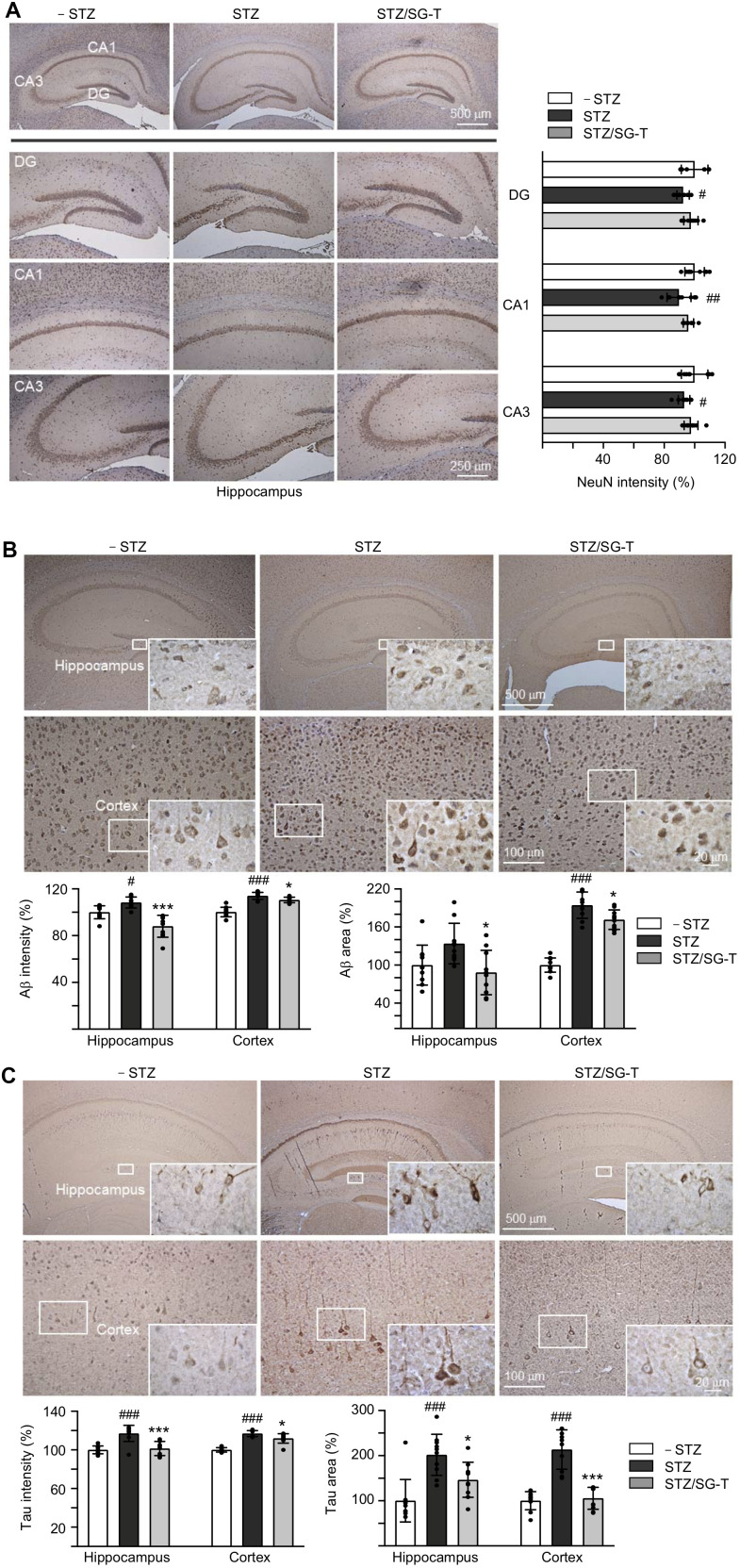
**Reduction of Aβ and Tau immunoreactivity of SG-Tang in STZ-treated 3×Tg-AD mice.** Mice in – STZ, STZ and STZ/SG-Tang groups received vehicle, STZ and STZ+SG-Tang, respectively during the course of the experiment. (**A**) Representative IHC images for NeuN and intensity quantification in the hippocampus of mice. DG, dentate gyrus; CA1 and CA3, *Cornu Ammonis* areas 1 and 3. (**B**, **C**) Representative IHC images for Aβ and Tau and intensity and area quantification in the hippocampus and cortex of mice. *P* values: comparisons between STZ vs. – STZ mice (^#^: *P* < 0.05, ^##^: *P* < 0.01, ^###^: *P* < 0.001), or STZ/SG-Tang vs. STZ mice (*: *P* < 0.05, ***: *P* < 0.001). (One-way ANOVA with a *post hoc* Tukey test).

Besides cognitive function, we examined NeuN (RNA binding protein, fox-1 homolog (*C. elegans*) 3), Aβ and Tau levels in 3×Tg-AD mice with STZ/SG-Tang treatment. In immunohistochemical analysis, STZ treatment reduced NeuN level in dentate gyrus (DG; 92%, *P* = 0.028) and *Cornu Ammonis* areas 1 (CA1; 90%, *P* = 0.003) and 3 (CA3; 93%, *P* = 0.043) of the hippocampus of 3×Tg-AD mice (STZ group). Meanwhile, SG-Tang treatment could mitigate this decrease to 96–98%, although not significantly (*P* > 0.05) (Figure 5A). However, STZ treatment increased expression of Aβ (intensity: 108–112%, *P* = 0.032–<0.001; area: 134–194%, *P* = 0.071–<0.001) in the hippocampus and cerebral cortex, whereas SG-Tang treatment reduced the levels of Aβ (intensity: 88–110%, *P* = 0.038–<0.001; area: 88–172%, *P* = 0.012) (STZ/SG-Tang group) (Figure 5B). Consistently, STZ treatment increased expression of Tau (intensity: 117%, *P* < 0.001; area: 202–214%, *P* < 0.001) in the hippocampus and cerebral cortex, whereas SG-Tang treatment normalized the levels of Tau (intensity: 101–112%, *P* = 0.011–<0.001; area: 146–106%, *P* = 0.023–<0.001) (Figure 5C). These results demonstrated the abnormal accumulations of Aβ and Tau in the hippocampus and cerebral cortex of STZ-treated 3×Tg-AD mice, and what is more SG-Tang treatment could reduce these important AD phenotypes.

### SG-Tang mitigated NLRP1 and NLRP3 expression in STZ-treated 3×Tg-AD mice

**Figure 6:**
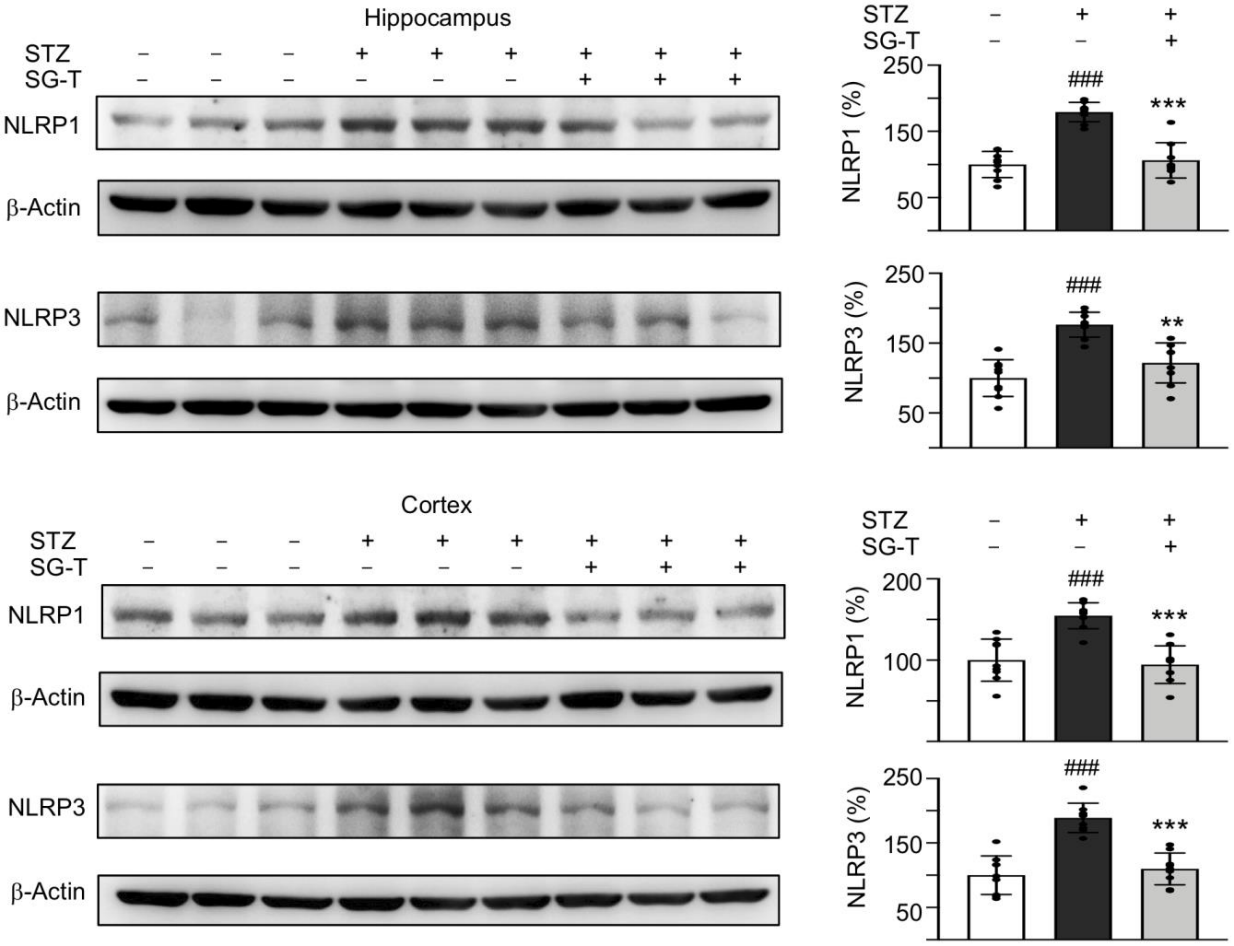
**Mitigation of NLRP1 and NLRP3 expression of SG-Tang in STZ-treated 3×Tg-AD mice.** Expression levels of NLRP1 and NLRP3 in hippocampus were analyzed by Western blot using β-Actin as a loading control. To normalize, the relative NLRP1 and NLRP3 of – STZ mice was set as 100%. *P* values: comparisons between STZ vs. – STZ mice (^###^: *P* < 0.001), or STZ/LSG-Tang vs. STZ mice (**: *P* < 0.01, ***: *P* < 0.001). (One-way ANOVA with a *post hoc* Tukey test).

We further examined the changes in inflammasome pathway in STZ-treated 3×Tg-AD mice (Figure 6). STZ application raised NLRP1 (179–155%, *P* < 0.001) and NLRP3 (177–189%, *P* < 0.001) levels in the hippocampus and cerebral cortex of 3×Tg-AD mice (STZ group), whereas SG-Tang treatment normalized these abnormal up-regulations to 106–95% in NLRP1 (*P* < 0.001) and 122–110% in NLRP3 (*P* = 0.001–<0.001). These results demonstrated the potential of SG-Tang to reduce neuroinflammation *in vivo*.

## DISCUSSION

Accumulated evidence has shown misfolded proteins aggregates as a trigger for chronic inflammation and neurodegeneration [34]. Aβ can bind to several innate immune receptors present on microglia [14, 15, 35], leading to generation of pro-inflammatory mediators [16, 17]. The paracrine effects of these mediators further affect neurite outgrowth by activating inflammasome [36]. *P. lactiflora* and *G. uralensis*, the components of SG-Tang, have been used traditionally to alleviate oxidation, inflammation and strengthen cytoprotection. *P. lactiflora* or its active compound paeoniflorin has exerted the beneficial effects in rodent models relevant to AD [37–39], as well as a cell model for the spinocerebellar ataxia 3 (SCA3) [40]. *G. uralensis* has anti-inflammatory and anti-oxidative activities in macrophages and hepatocytes [41, 42].

SG-Tang has been used to inhibit chemokine activity in keratinocytes [43]. Here we find that SG-Tang demonstrates neuroprotection against Aβ aggregation and neuroinflammation, particularly targeting inflammasome, in cell and animal models of AD.

Inflammasomes are cytosolic protein complexes that promote the maturation and the secretion of pro-inflammatory mediators [44]. Reports have indicated the priming and activation of inflammasome receptors, such as NLRP1 and NLRP3, in neurons. NLRP1 inflammasome complex is up-regulated in rat cortical neurons after traumatic brain injury, stroke and hippocampal aging [45–49]. Up-regulation of NLRP1 in cortical neurons further activates caspase 1, enhances Aβ production and axonal degeneration [36]. In the APP/PS1 mouse model of AD, the activation of NLRP3 induces the production of IL-1β and IL-6 [50, 51]. Knockout of NLRP3 on APP/PS1 mice reduces impairment of spatial memory and enhances Aβ clearance [52]. Our results also demonstrated that pro-inflammatory cytokines in CM/+IFN-γ potentiated the up-regulation of iNOS, NLRP1, NLRP3, TNF-α, IL-1β, IL-6 and caspase-1 activity, as well as impairment of neurite outgrowth by Aβ overexpression, while SG-Tang treatment normalized the expressions of NLRP1/NLRP3 pathways and improved the neurite outgrowth (Figure 3). In STZ-treated 3×Tg-AD, SG-Tang treatment further improved working and spatial memories (Figure 4), reduced abnormal accumulations of Aβ and Tau (Figure 5), as well as down-regulated NLRP1/NLRP3 (Figure 6). These findings further support the potentials of SG-Tang as NLRP1/NLRP3 inhibitors for treating AD.

The NLRP3 inflammasome is activated by ATP and certain bacterial toxins [53]. The activation of NLRP3 pathway can be a two-step process. In priming, the expression of NLRP3, caspase-1 and pro-IL-1β are increased. This transcriptional up-regulation can be induced through engaging TLRs [54], or through pro-inflammatory cytokines [55]. Upon activation, NLRP3 causes proteolytic production of active caspase-1, which leads to conversion of IL-1β and IL-18 inactive precursors into their mature, active forms [56, 57]. It has been shown that Aβ could directly interact with NLRP3, leading to the activation of the NLRP3 [58]. Our results further demonstrated that Aβ also up-regulated the expression of NLRP3 in the neuronal cells differentiated from SH-SY5Y, indicating its priming effect on inflammasome, and SG-Tang could normalize the priming and activation of NLRP3 in neurons.

Our results also showed that Aβ and CM/+IFN-γ up-regulated the expression of NLRP1 (Figure 3). Inflammasome complex consisting of NLRP1 and the apoptosis-associated speck-like protein (ASC) can also recruit and activate caspase-1 [56]. NLRP1 can be activated by anthrax lethal toxin [59–61]. Interestingly, cerebral NLRP1 levels in APP/PS1 AD mice are up-regulated, while knockdown of NLRP1 can improve cognitive functions [62]. Our results suggested that Aβ and CM/+IFN-γ could upregulate the expression of NLRP1, while SG-Tang normalized the up-regulation of NLRP1. Further study will be warranted to identify the activators of NLRP1 in CM/+IFN-γ, as well as the regulatory mechanisms of inflammasome by SG-Tang.

IFN-γ, a cytokine critical for innate and adaptive immune responses against viral and protozoal infections, activates HMC3 to release pro-inflammatory cytokines, including TNF-α, IL-1β and IL-6 (Figure 2), all of them are important transcriptional regulator of inflammasome pathways. In murine macrophages, TNF-α induces NLRP3 expression and thus priming the NLRP3 inflammasome for subsequent activation [63]. Overexpression of TNF-α in 3×Tg-AD mice enhances intracellular levels of Aβ and Tau, as well as learning and memory deficits [64]. Inhibition of TNF-α can reduce cognitive deficits induced by Aβ [65]. Therefore, the high concentration of TNF-α in CM/+IFN-γ could activate NLRP3 in our neuronal cells differentiated from SH-SY5Y cells (Figure 3B). IFN-γ also regulates the secretion of IL-1β [66], which further induces expression of TNF-α [67], iNOS and release of NO [68]. On the other hand, the maturation of IL-1β is tightly controlled by NLRP3 [56]. IL-6, a pleiotropic cytokine, regulates inflammation in inflammasome-independent manner [51]. However, blockage of IL-6 signaling blunts the activation of NLRP3 in diabetic C57BL/KsJ-db/db mice [69]. Therefore, the high level of IL-6 in CM/+IFN-γ could also contribute to the up-regulation of NLRP3 inflammasome pathway in neuronal cells differentiated from SH-SY5Y cells (Figure 3B).

Two main active components, paeoniflorin and ammonia glycyrrizinate, have been identified in SG-Tang [23]. Paeoniflorin is known to exhibit a beneficial therapeutic effect via reducing neuroinflammation in APP/PS1 and PS2 AD mice [38, 39]. It also exerts anti-aggregation effect on SCA3 model [40]. Glycyrrizinate can reduce activation of microglia by Aβ [70]. In SCA3 cell model, it further demonstrates neuroprotective potentials against aggregation formation and upregulates anti-oxidative pathway [71]. Both paeoniflorin and glycyrrizinate are capable of crossing the blood–brain barrier (BBB) [72], suggesting that these two constituents of SG-Tang may employ potentials against aggregation and neuroinflammation by crossing BBB of 3×Tg-AD mice.

The transgenic expressions of APP/Tau and hyperglycemia in 3×Tg-AD mice last the depositions of Aβ/Tau, neuroinflammation and neurodegeneration. Therefore, it is possible that sustained SG-Tang treatment is necessary to attenuate the neurodegeneration, whereas short-term exposure of SG-Tang is not likely to demonstrate neuroprotective effects in this AD model. Future study will be warranted to confirm the temporal therapeutic window of SG-Tang treatment in AD.

## CONCLUSION

In this study, we have provided evidence that NLRP1/NLRP3 inflammasome pathways can be up-regulated by microglia-derived pro-inflammatory factors and Aβ overexpression. SG-Tang could serve as a neuroprotective strategy against Aβ aggregation and neuroinflammation via down-regulating the NLRP1/NLRP3 pathways. Our results consolidate the role of microglia-mediated neuroinflammation in AD pathogenesis, impacting the treatment for AD targeting inflammasome. Future work with large sample sizes will be warranted to strengthen the conclusions and uncover the main constituents and more mechanisms of the neuroprotective effects of SG-Tang.

## MATERIALS AND METHODS

### Test compound and formulated Chinese herbal medicine

The formulated CHM SG-Tang (Code: 0703H, Sun Ten Pharmaceutical, New Taipei City, Taiwan) was made of *P. lactiflora* and *G. uralensis* at 1:1 (w/w) ratio [23]. The ingredients *P. lactiflora* and *G. uralensis* are collected from An Hui and Inner Mongolia, China, respectively [25] and the chemical identities of these plant materials have been characterized [73]. SG-Tang stock solution was prepared by dissolving 5 g powder in 10 ml ddH_2_O. The supernatant was collected following centrifugation at 4000 rpm at room temperature for 10 min.

### Cell culture

The Dulbecco’s modified Eagle medium/Ham’s nutrient mixture F12 (DMEM/F12) containing 10% fetal bovine serum (FBS) (Thermo Fisher Scientific, Waltham, MA, USA) was used to culture human HMC3 microglial cell line (ATCC CRL-3304) and human Aβ-GFP neuroblastoma SH-SY5Y cell line [27]. Blasticidin (5 μg/ml, InvivoGen, San Diego, CA, USA) and hygromycin (100 μg/ml, InvivoGen) were added to the medium to select cells with Aβ-GFP transgene, while doxycycline (5 μg/ml, Sigma-Aldrich, St. Louis, MO, USA) was added to induce expression of Aβ-GFP.

### High content analysis of Aβ-GFP fluorescence and oxidative stress

Aβ-GFP SH-SY5Y cells (2.5 × 10^4^) were seeded into a 96-well plate with retinoic acid (10 μM; Sigma-Aldrich) on day 1 [74]. On the next day, curcumin (1.2–5 μM; Sigma-Aldrich), a potent inhibitor against Aβ aggregations as a positive control [75], or SG-Tang (1–100 μg/ml) were added for 8 h. Doxycycline (5 μg/ml) were added to induce Aβ-GFP expression for another 7 days. Then cells stained with Hoechst 33342 (0.1 μg/ml, Sigma-Aldrich) were captured by Micro Confocal High-Content Imaging System (Molecular Devices, Synnyvale, CA, USA) at excitation/emission wavelengths of 482/436 nm, and analyzed by ImageXpress (Molecular Devices). To measure ROS, cells were incubated with the reddish fluorogenic CellROX reagent (5 μM; Molecular Probes) and Hoechst 33342 at 37° C for 30 min. Micro Confocal High-Content Imaging System at excitation/emission wavelengths of 640/665 nm and ImageXpress were used for the acquisition and analysis of cell images.

### Real-time PCR analysis of Aβ-GFP RNA

Total RNA was reverse transcribed by SuperScript III reverse transcriptase (Invitrogen, Waltham, MA, USA). One hundred ng cDNA and the gene-specific TaqMan fluorogenic probes PN4331348 (EGFP) and 4326321E (HPRT1) were used for real-time PCR by StepOnePlus Real-time PCR system (Applied Biosystems, Foster City, CA, USA). Fold change of Aβ-GFP expression was evaluated by calculate 2^ΔCt^, in which C_T_ indicates the cycle threshold and ΔC_T_ = C_T_ (HPRT1) − C_T_ (EGFP).

### High content analysis of neurite outgrowth

Cells were fixed by 4% paraformaldehyde (Sigma Aldrich) for 15 min, permeabilized by 0.1% Triton X-100 (Sigma-Aldrich) for 10 min, blocked by 3% bovine serum albumin (BSA, Sigma-Aldrich) for 20 min, and stained by anti-TUBB3 (neuronal class III β-tubulin) (1:1000; Covance, Princeton, NJ, USA) antibody at 4° C overnight. The cells were washed by phosphate-buffered saline (PBS) for twice and stained with the secondary Alexa Fluor ®555 goat anti-rabbit antibody (1:1000; Molecular probes) at room temperature for 3 h, and with 4’-6-diamidino-2-phenylindole (DAPI, 0.1 μg/ml, Sigma-Aldrich) for 30 min. Images of neurites were captured by Micro Confocal High-Content Imaging System (Molecular Devices), and analyzed by MetaXpress Neurite Ougrowth Application Module (Molecular Devices).

### Activation of HMC3 microglia and detection of inflammatory mediators

HMC3 cells (2 × 10^5^) were seeded into a well of 6-well dishes for 24 h. IFN-γ (100 ng/ml) (PeproTech, Rocky Hill, NJ, USA) were added for 24 h to activate microglia. The level of NO in fresh cell culture medium was evaluated by Griess assay (Thermo Fisher Scientific). Human Instant enzyme-linked immunosorbent assay (ELISA)^™^ Kit (Invitrogn) was used to determine the levels of IL-1β, IL-6 and TNF-α, in medium. The culture medium with or without inflammatory factors (CM/±IFN-γ, conditioned medium activated by IFN-γ or not) was centrifuged and stored at -80° C.

After treatment with IFN-γ, HMC3 cells were also fixed, permeabilized, and stained with anti-CD68 (CD68 molecule, 1:1000; Cell Signaling*,* Danvers, MA, USA) or anti-MHCII (major histocompatibility complex class II, 1:1000; Invitrogen) antibodies at 4° C overnight. Cells were washed twice by PBS, and stained with Alexa Fluor 555-donkey anti-rabbit or Cy^TM^5-goat anti-mouse secondary antibody (1:1000; Invitrogen) for 2 h at room temperature, and DAPI (0.1 μg/ml) for 30 min. Zeiss LSM 880 confocal laser scanning microscope (Zeiss, Oberkochen, Germany) was used to capture the fluorescent cell images.

### Neuroinflammation induction in Aβ-GFP SH-SY5Y cells

To induce neuroinflammation in Aβ-GFP SH-SY5Y cells, retinoic acid was removed and CM/+IFN-γ was added at a 1:1 ratio in the last two days. The collected CM/**-**IFN-γ was also added to uninduced and untreated cells for comparison. On day 8, cells were fixed, permeabilized, stained with primary/secondary antibodies for neurite outgrowth analysis as described. ROS was also assayed.

### Caspase-1 activity assay

Caspase-1 activity in cells was examined by ICE fluorometric assay kit (BioVision, Milpitas, CA, USA), with FLx800 fluorescence microplate reader (Bio-Tek) at 400/505 nm for excitation/emission.

### Western blot

Total proteins were prepared using lysis buffer containing 50 mM Tris-HCl pH8.0, 1% Triton X-100, 0.1% SDS, 1 mM EDTA pH8.0, 1 mM EGTA pH8.0, 150 mM NaCl, 0.5% sodium deoxycholate, and protease inhibitor cocktail (Sigma-Aldrich). Proteins (20 μg) were separated on 10% SDS-PAGE and blotted to polyvinylidene difluoride (PVDF) membranes (Sigma-Aldrich). The membrane was blocked by 3% BSA for 20 min, probed with anti-iNOS (1:500; Cell Signaling, Danvers, MA, USA), anti-NLRP1 (1:500; Novus Biologicals, Centennial, CO, USA), anti-NLRP3 (1:500; Cell Signaling), anti-TNF-α (1:1000; Abcam, Cambridge, MA, USA), anti-IL-1β (1:1000; Abcam), anti-IL-6 (1:1000; Abcam), or anti-GAPDH (glyceraldehyde-3-phosphate dehydrogenase) (1:1000; MDBio Inc., Taipei, Taiwan) antibodies. After washed twice with PBS, the membrane was treated with horseradish peroxidase-conjugated goat anti-mouse or anti-rabbit IgG antibody (1:5000; GeneTex, Irvive, CA, USA) and the chemiluminescent substrate (Millipore).

### Animal studies

Mice harboring APP_Swe_, presenilin 1 (PS1)_M146V_ and microtubule associated protein tau (Tau)_P30IL_ transgenes (3×Tg-AD, 004807) [28], were purchased from the Jackson Laboratory (Bar Harbor, ME, USA). Mice were maintained at 20–25° C and 60% relative humidity under a daily light/dark (12 h/12 h) cycle in the Animal House Facility of National Taiwan Normal University (NTNU), Taipei, Taiwan. Four-month-old mice were randomly assigned to 3 groups: no treatment, treatment with STZ, and treatment with STZ/SG-Tang (*n* = 10 in each group). To accelerate the development of AD phenotypes [33], the mice fasted for 12 h were intraperitoneally (i.p.) injected with STZ (100 mg/kg; Sigma-Aldrich) or vehicle (0.1 M sodium citrate pH4.5) as previously described [76]. SG-Tang (0.4%) was added to drinking water for 14 weeks (from day -60 to day 38) in STZ/SG-Tang group. Mouse body weight and blood glucose level were measured every week. All animal procedures, followed the ARRIVE (Animal Research: Reporting *In Vivo* Experiments) guidelines, were approved by the Institutional Animal Care and Use Committee of NTNU (Permit Number: 103002).

### Behavioral analyses

To conduct the open field test, the mouse was placed in the center of an open-field box (30 cm long, 30 cm high, and 30 cm wide) to freely explore the box for 10 min. The routes were recorded by a video camera mounting on the ceiling above the box, and analyzed by PhenoTracker (TSE system, Thuringia, Germany).

For the Y-maze, the mouse was placed in one of the arm compartments (40 cm long, 30 cm high, and 15 cm wide) for 8 min. The spontaneous alternation behavior, used to assess spatial working memory in mice [77], was defined as the percentage of actual to possible alternations.

The water maze apparatus was composed of a white-opaque painted circular pool (diameter 100 cm and height 76 cm) with a submerged platform (1 cm below the water surface) and 4 cues providing landmarks in the testing room. The pool was filled up with water (24–26° C, 35 cm high). For pretraining, the mouse was placed in the pool for 60 sec. After three trials of pretraining, the mouse was placed on the platform in the center of the pool for 20 sec. For training, the platform was placed in a quadrant with a cue. The mouse was placed in the pool semi-randomly. The trial ended either when the mouse climbed onto the platform or when 60 seconds had elapsed, and then the mouse was placed on the platform and faced the cue for 20 sec. Four training trials were applied for 4 days. Three testing trials were given to the mouse to assess the time to climb onto the platform. The probe trials, by putting the mouse to the pool with no the platform for one min, were given 48 h later to record the time spent in the target quadrant of previous platform. The data were collected by a video camera suspended 250 cm above the center of the pool, and analyzed by PhenoTracker.

### Immunohistochemistry and image analysis

Mouse brains were fixed in 4% paraformaldehyde overnight, and cryoprotected in 30% sucrose at 4° C. Brain sections (30 μm) were coronally cut by Leica RM2125 RTS cryostat (Leica, Wetzlar, Germany). Heat-induced antigen retrieval for immunohistochemistry (IHC) was performed using antigen retrieval buffer (Thermo Fisher Scientific). Brain sections were pretreated with 1% H_2_O_2_ for 15 min, and then incubated with anti-NeuN, anti-Aβ, or anti-Tau antibodies (1:100; Bioss Inc., Woburn, MA, USA) overnight at 4° C. The sections were washed twice by PBS. The bindings of antibodies were detected by UltraVision™ Quanto detection system (Thermo Fisher Scientific). The sections were also stained with hematoxylin (Thermo Fisher Scientific), dehydrated by ethanol and xylene (Sigma-Aldrich), and mounted by Micromount (Leica Biosystems, Wetzlar, Germany). All image analysis were performed using IHC toolbox plugin of ImageJ [78].

### Statistical analysis

All quantitative data were presented as the mean ± standard deviation. Three independent tests in two or three biological replicates were performed in each experiment. Differences between groups were compared by two-tailed Student’s *t* test or one-way or two-way analysis of variance (ANOVA) with a *post hoc* Tukey test. *P* values < 0.05 were statistically significant.
